# Significant muscle loss after stereotactic body radiotherapy predicts worse survival in patients with hepatocellular carcinoma

**DOI:** 10.1038/s41598-022-21443-6

**Published:** 2022-11-09

**Authors:** Jen-Fu Yang, Wen-Yen Huang, Cheng-Hsiang Lo, Meei-Shyuan Lee, Chun-Shu Lin, Po-Chien Shen, Yang-Hong Dai, Ying-Fu Wang, Teng-Wei Chen

**Affiliations:** 1 Department of Radiation Oncology, Tri-Service General Hospital, National Defense Medical Center, Taipei, Taiwan; 2 Institute of Clinical Medicine, National Yang-Ming University, Taipei, Taiwan; 3 School of Public Health, National Defense Medical Center, Taipei, Taiwan; 4 Graduate Institute of Clinical Medicine, Taipei Medical University, Taipei, Taiwan; 5 Division of General Surgery, Department of Surgery, Tri-Service General Hospital, National Defense Medical Center, No. 325, Sec. 2, Cheng-Kong Rd., Nei-Hu, Taipei, 11490 Taiwan

**Keywords:** Gastroenterology, Medical research, Oncology

## Abstract

The relationship between sarcopenia and treatment outcomes, especially in patients with hepatocellular carcinoma (HCC) undergoing stereotactic body radiotherapy (SBRT) has not been well-explored. This study aimed to investigate the effects of sarcopenia on the survival and toxicity after SBRT in patients with HCC. We included 137 patients with HCC treated with SBRT between 2008 and 2018. Sarcopenia was defined as a skeletal muscle index (SMI) of < 49 cm^2^/m^2^ for men and < 31 cm^2^/m^2^ for women using computed tomography images at the mid-level of the third lumbar vertebra. The SMI change was presented as the change per 90 days. The Kaplan–Meier method was used for survival estimation, and the Cox regression was used to determine prognosticators. Sarcopenia was present in 67 of 137 eligible patients. With the median follow-up of 14.1 months and 32.7 months in the entire cohort and in those alive, respectively, patients with pre-SBRT sarcopenia or SMI loss ≥ 7% after SBRT had worse overall survival than their counterparts. Significant survival predictors on multivariate analysis were SMI loss ≥ 7% after SBRT [hazard ratio (HR): 1.96, p = 0.013], presence of extrahepatic metastasis (HR: 3.47, p < 0.001), neutrophil-to-lymphocyte ratio (HR: 1.79, p = 0.027), and multiple tumors (HR: 2.19, p = 0.003). Separate Cox models according to the absence and presence of pre-SBRT sarcopenia showed that SMI loss ≥ 7% remained a significant survival predictor in patients with sarcopenia (HR: 3.06, p = 0.017) compared with those without sarcopenia. SMI loss ≥ 7% is also a predictor of the Child–Pugh score increase by ≥ 2 points after SBRT. SMI loss ≥ 7% after SBRT is a significant prognostic factor for worse survival and is associated with liver toxicity compared with pre-SBRT sarcopenia.

## Introduction

Hepatocellular carcinoma (HCC) is the sixth most common malignancy worldwide and the fourth most common cause of cancer-related death^1^. For decades, new advances in the management of HCC have improved patients’ clinical outcomes. The emergence of stereotactic body radiotherapy (SBRT) has expanded the clinical indications for radiotherapy (RT) in HCC treatments. For inoperable or locally advanced HCC, SBRT provides favorable local control and survival^2–4^. Although SBRT has shown efficacy, the prognosis remains unsatisfactory in certain subgroups with considerable intra- or extra- hepatic progression. To refine outcome assessment and patient selection for SBRT, the identification of potential prognostic factors in HCC is of clinical importance.

Sarcopenia, accompanied by progressive and generalized loss of muscle mass and function, is known to result in physical disability, poor quality of life, and mortality^5^. Sarcopenia can develop primarily because of aging or secondarily as a consequence of chronic diseases and malignancy^5,6^. More recently, sarcopenia has been of increasing interest in oncology because of its high prevalence and association with adverse outcomes in a wide range of cancers^7–13^, including HCC. The incidence of sarcopenia in HCC is relatively high, probably because frequently associated liver cirrhosis, the underlying metabolic changes of which contribute to the occurrence of sarcopenia. Previous reports have shown sarcopenia is a predictor of worse outcomes in liver cirrhosis patients^14,15^. Thus, sarcopenia may have a greater impact on HCC treatment. Several studies have demonstrated its prognostic effect on HCC in various clinical settings^16–23^, such as posthepatectomy^19,20^, post-transcatheter arterial chemoembolization^21^, post-sorafenib^22^, and post-lenvatinib^23^.

Regarding the relationship between sarcopenia and treatment outcomes, there are no published reports that specifically focus on patients with HCC undergoing SBRT. We hypothesized that sarcopenia would result in poor outcomes in this cohort. The primary objective of the present study was to investigate the impact of pre-SBRT sarcopenia and muscle loss on outcomes and toxicity after SBRT for HCC.

## Methods

### Patients

This study has been approved by the Institutional Review Board of Tri-Service General Hospital (Protocol code: TSGH-1-107-05-016). As a waiver of informed consent was obtained, we retrospectively reviewed the medical charts of patients with HCC treated with SBRT between January 2008 and December 2018. The study protocol was performed in accordance with the ethical guidelines of the Declaration of Helsinki. The diagnosis of HCC was based on either characteristic features on one or two imaging studies or histological confirmation. The eligibility criteria were as follows: (1) Unresectable or medically inoperable HCC via a multidisciplinary team discussion. (2) No previous hepatic irradiation. (3) Eastern Cooperative Oncology Group (ECOG) performance status of 0 to 2. (4) Any Child–Pugh (CP) class. (5) Patients with at least one set of abdominal computed tomography (CT) before and after SBRT.

### SBRT technique

The detailed SBRT techniques, dose prescription, and constraints for dose-limiting organs have been described in our previous publication^24^. Briefly, SBRT was administered using CyberKnife (Accuray, Sunnyvale, CA, USA) with tumor tracking devices until June 2017. Subsequently, SBRT was administered using Versa HD with an Active Breathing Coordinator (Elekta AB, Stockholm, Sweden). If patients were unable to hold their breath, abdominal compression was used to minimize liver motion, and 4-dimensional CT was used to generate the internal target volume. Fiducial marker implantation before treatment was strongly recommended for image guidance. The gross tumor volume, which was coincident with the clinical target volume, was defined as the visible tumor on contrast CT or magnetic resonance imaging (MRI). There was a 0–8 mm expansion around the clinical target volume to create the planning target volume. Margin modification was acceptable for respecting dose-limiting organs.

### Evaluation and follow up

All patients underwent evaluations including physical examination, complete blood counts, serum biochemistry test, and alpha fetal protein test. Surveillance MRI and/or CT of the abdomen were generally ordered 1–3 months after completion of SBRT with 3-to-4-month intervals thereafter.

For liver toxicity assessment, we recorded an increase in CP score by ≥ 2 within 3 months of SBRT completion in the absence of hepatic tumor progression, according to modified RECIST criteria, version 1.1^25^. Patients included in the toxicity analysis were supposed to have adequate follow-up of 3 months, death and/or occurrence of toxicity within 3 months, and evaluable laboratory data.

### Assessment of sarcopenia

**Figure 1:**
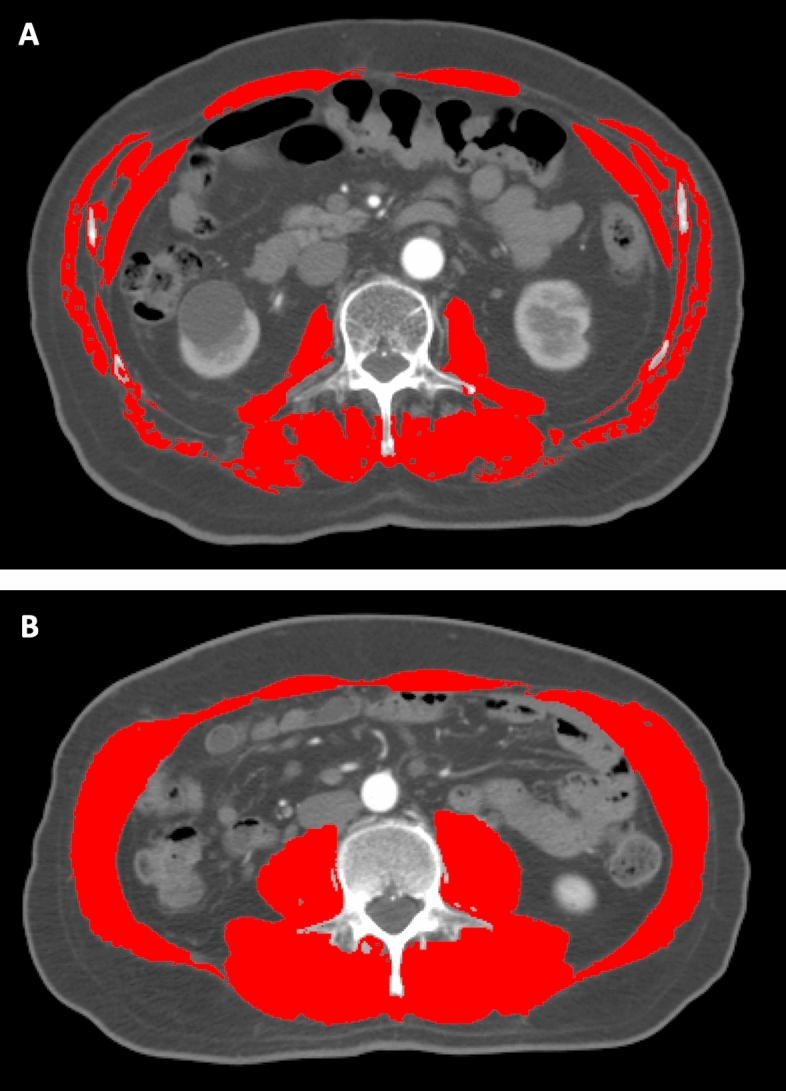
Illustrations of areas of skeletal muscle mass on computed tomography at the mid-level of the third lumbar vertebrate (**A**) patients with sarcopenia, (**B**) patients without sarcopenia.

Sarcopenia was assessed using the L3-skeletal muscle index (SMI) method. Two sets of CT images were acquired, one before SBRT and the other after SBRT. The single slice CT at the mid-level of the third lumbar vertebrae was chosen for muscle measurement because the muscle mass at this level represents the surrogate parameters of whole-body muscle mass^26,27^. Regions of interest, including the psoas, paraspinal, transversus abdominis, rectus abdominis, and internal and external oblique muscles were quantified within a range of − 29 to + 150 Hounsfield units and outlined manually on the CT images (Fig. 1). Cross-sectional areas (in cm squared) of the sum of these targeted muscles were calculated using SliceOmatic software (version 5.0; TomoVision). The SMI was reported as a normalizing measured area with a square of height. Two radiation oncologists, Jen-Fu Yang and Po-Chien Shen were trained to correctly identify the muscles and segment each selected CT image in a blinded fashion to obtain a mean SMI.

Skeletal muscle changes were assessed by comparing the SMI difference between the two chosen CT slices. Considering the inconsistent scan interval in each patient, SMI change was standardized as the change per 90 days. The SMI cut-off values presented in a Korean study were chosen to evaluate our patients, which define sarcopenia as SMI ≤ 49 cm^2^/m^2^ for men and ≤ 31 cm^2^/m^2^ for women^28^. We considered this definition derived from a similar population in terms of geography and ethnicity to be more applicable to the Taiwanese population. Given the diverse definitions that are currently available, we also incorporated other definitions^29–31^ into our analysis to strengthen the results.

### Statistics

Comparisons between groups were performed using the Pearson’s chi-squared test or Fisher’s exact test for categorical variables and the Student’s t-test or Mann–Whitney U test for continuous variables. The optimal cut-off point for continuous variables was estimated using a receiver operating characteristic (ROC) curve. Inter-observer reliability was assessed by computing the intraclass correlation coefficients. Overall survival (OS) was measured from the commencement day of SBRT to the date of death or last follow-up. Progression free survival (PFS) was measured from the commencement day of SBRT to the date of tumor progression at any sites or death or last follow-up. All survival analysis were conducted using the Kaplan–Meier method and compared using the log-rank test. Univariate and multivariate Cox proportional hazards regression models were used to determine the potential prognostic factors associated with survival. To identify the predictors of SMI change, a logistic multivariable regression model was applied with variance inflation factor calculation to detect multicollinearity among variables. The significance level was set at a global 2-tailed p-value of < 0.05, except a p-value of < 0.10 was selected when deciding which variable in a univariate Cox model was introduced into a multivariate Cox model. SPSS (SPSS Inc. Chicago, IL, USA) version 22 was used for all the data analyses.

## Results

### Patient characteristics

**Figure 2:**
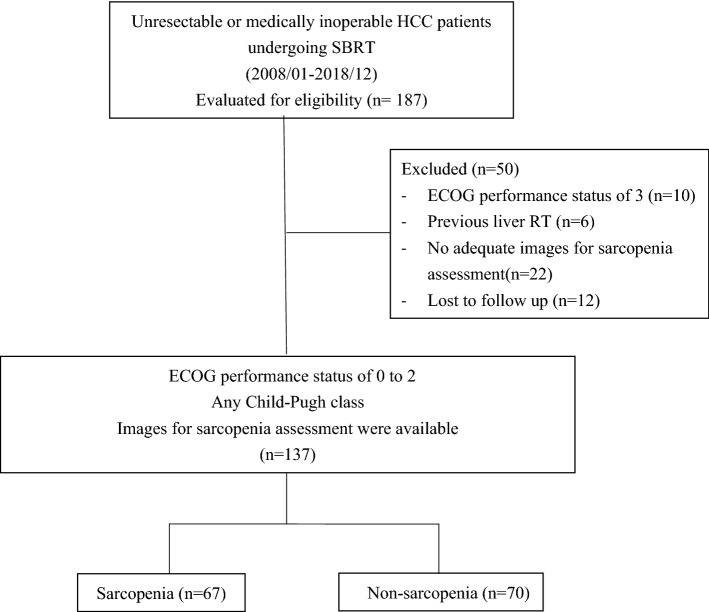
Flow diagram detailing inclusion and exclusion of patients.

In total, 187 patients with HCC were initially identified by chart review, of which 50 patients were excluded due to ineligibility or loss to follow-up after RT (Fig. 2). The remaining 137 patients were included in the final analysis. The comparison of patients’ clinicopathological features according to sarcopenia status before SBRT is listed in Table 1. Overall, 67 (49%) patients had sarcopenia, and 70 (51%) patients did not have sarcopenia. Patients with sarcopenia were more likely to be males or have a larger tumor size, and lower body mass index.

**Table 1:** Patient characteristics according to the presence of pre-SBRT sarcopenia.

Variable	No. (%)			P-value
	Overall N = 137	Sarcopenia N = 67	Non-sarcopenia N = 70	
**Age, year**				
Mean (SD)*	63.8 (12.7)	65.1 (11.7)	62.6 (13.5)	0.24
≤ 60	52 (38.0)	23 (34.3)	29 (41.4)	0.39
> 60	85 (62.0)	44 (65.7)	41 (58.6)	
**Sex**				< 0.001
Male	106 (77.8)	63 (94.0)	43 (61.4)	
Female	31 (22.2)	4 (6.0)	27 (38.6)	
**Liver disease**				0.80
HBV	71 (51.8)	37 (55.2)	34 (48.6)	
HCV	36 (26.3)	16 (23.9)	20 (28.6)	
HBV and HCV	8 (5.8)	3 (4.5)	5 (7.1)	
Non-virus	22 (16.1)	11 (16.4)	11 (15.7)	
**ECOG**				0.27
0–1	119 (86.9)	56 (83.6)	63 (90.0)	
2	18 (13.1)	11 (16.4)	7 (10.0)	
**AFP, ng/ml**				0.97
≤ 200	79 (57.7)	40 (59.7)	39 (55.7)	
> 200	51 (37.2)	26 (38.8)	25 (35.7)	
missing	7 (5.1)	1 (1.5)	6 (8.6)	
**ALBI score**				0.51
Mean (SD)*	− 2.42 (0.57)	− 2.39 (0.55)	− 2.45 (0.60)	
**Child–Pugh class**				0.85
A	110 (80.3)	55 (82.1)	55 (78.6)	
B	23 (16.8)	10 (14.9)	13 (18.6)	
C	4 (2.9)	2 (3.0)	2 (2.9)	
**Albumin**				0.42
Mean (SD)*	3.70 (0.56)	3.66 (0.56)	3.74 (0.56)	
**NLR**				0.23
≤ 2.5	57 (41.6)	25 (37.3)	32 (45.7)	
> 2.5	62 (45.3)	34 (50.7)	28 (40.0)	
Missing	18 (13.1)	8 (11.9)	10 (14.3)	
**Prior treatment**				0.12
Yes	85 (62)	46 (68.7)	39 (55.7)	
No	52 (38)	21 (31.3)	31 (44.3)	
**No. of tumor**				0.69
Multiple	78 (56.9)	37 (55.2)	41 (58.6)	
Single	59 (43.1)	30 (44.8)	29 (41.4)	
**Tumor size, cm**				0.05
≤ 5	67 (48.9)	27 (40.3)	40 (57.1)	
> 5	70 (51.1)	40 (59.7)	30 (42.9)	
**Macrovascular invasion**				> 0.99
Yes	47 (34.3)	23 (34.3)	24 (34.3)	
No	90 (65.7)	44 (65.7)	46 (65.7)	
**Extrahepatic metastasis**				0.93
Yes	27 (19.7)	13 (19.4)	14 (20.0)	
No	110 (80.3)	54 (80.6)	56 (80.0)	
**BMI**				< 0.001
Mean (SD)*	24.7 (4.1)	23.2 (3.2)	26.1 (4.5)	
**SMI**				< 0.001
Mean (SD)*	44.9 (8.8)	40.8 (5.5)	48.8 (9.5)	
**BED, Gy**				0.30
Mean (SD)*	87.4 (21.9)	85.4 (23.5)	89.4 (20.1)	

SBRT: stereotactic body radiotherapy; HBV: hepatitis B virus; HCV = hepatitis C virus; ECOG: Eastern Cooperative Oncology Group; AFP: alpha fetal protein; ALBI = albumin-bilirubin; NLR = neutrophil lymphocyte ratio; BMI: body mass index; SMI: skeletal muscle index; BED: biological effective dose; SD: standard deviation. *t-test.

The intraclass correlation coefficients for SMI (pre-SBRT: 0.99; post-SBRT: 0.99) reflect the high consistency of measurements between the two observers. The median interval between pre- and post-SBRT CT scans was 3 (interquartile range, 2.1–3.7) months. For 90 days, patients with sarcopenia had a mean SMI loss of 1.9, with a relative loss of 4.5%, while patients without sarcopenia had a mean SMI loss of 1.3, with a relative loss of 2.2%. Through ROC curve estimation, 7% was identified to best express the discriminatory ability of SMI for survival. eTable 1 in the Supplement revealed clinicopathological characteristics of patients with or without SMI loss ≥ 7%.

### Survival

**Figure 3:**
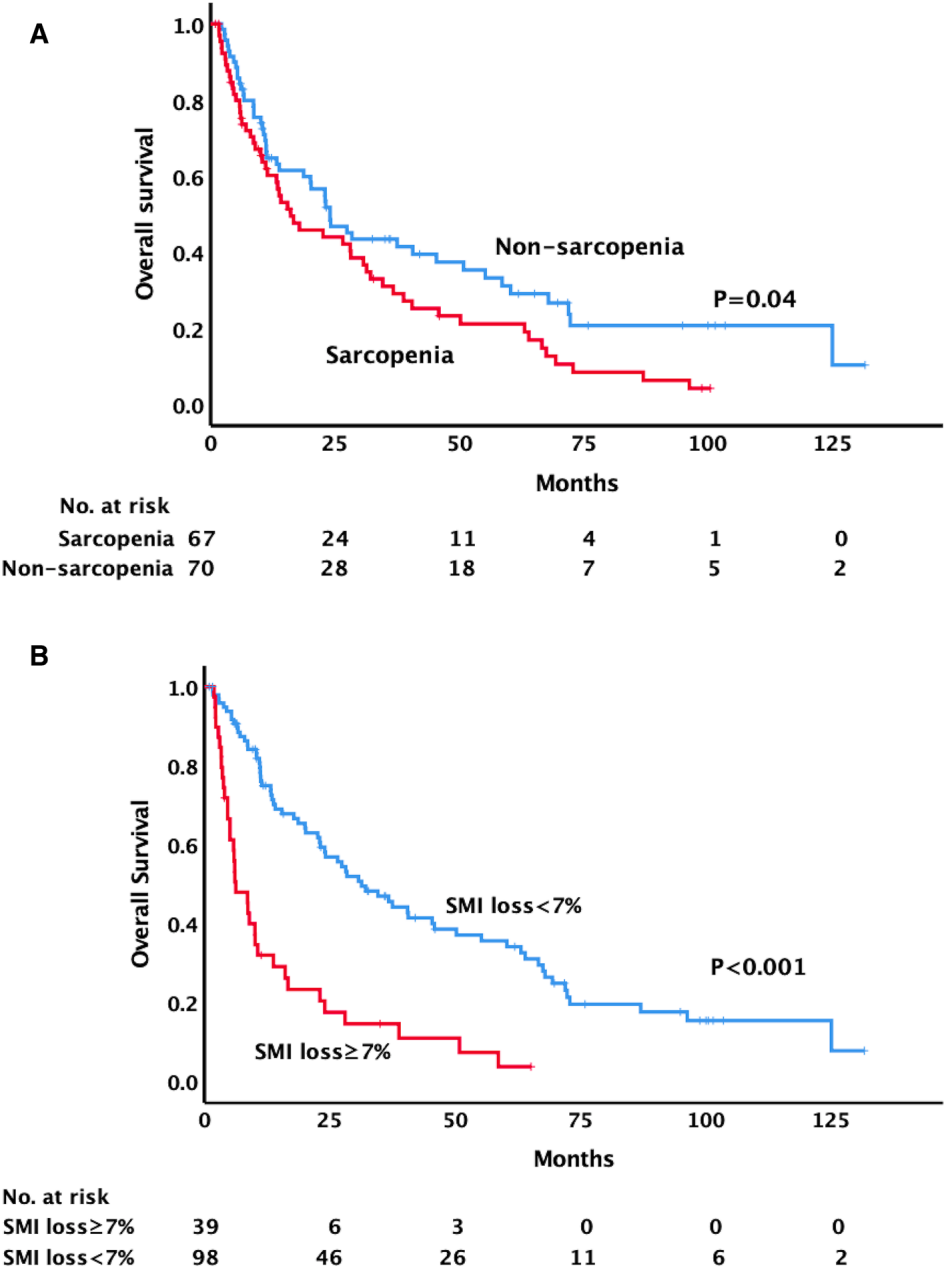
Overall survival comparisons between (**A**) patients with and without pre-SBRT sarcopenia (**B**) patients with and without skeletal muscle loss after SBRT using Kaplan–Meier method.

**Figure 4:**
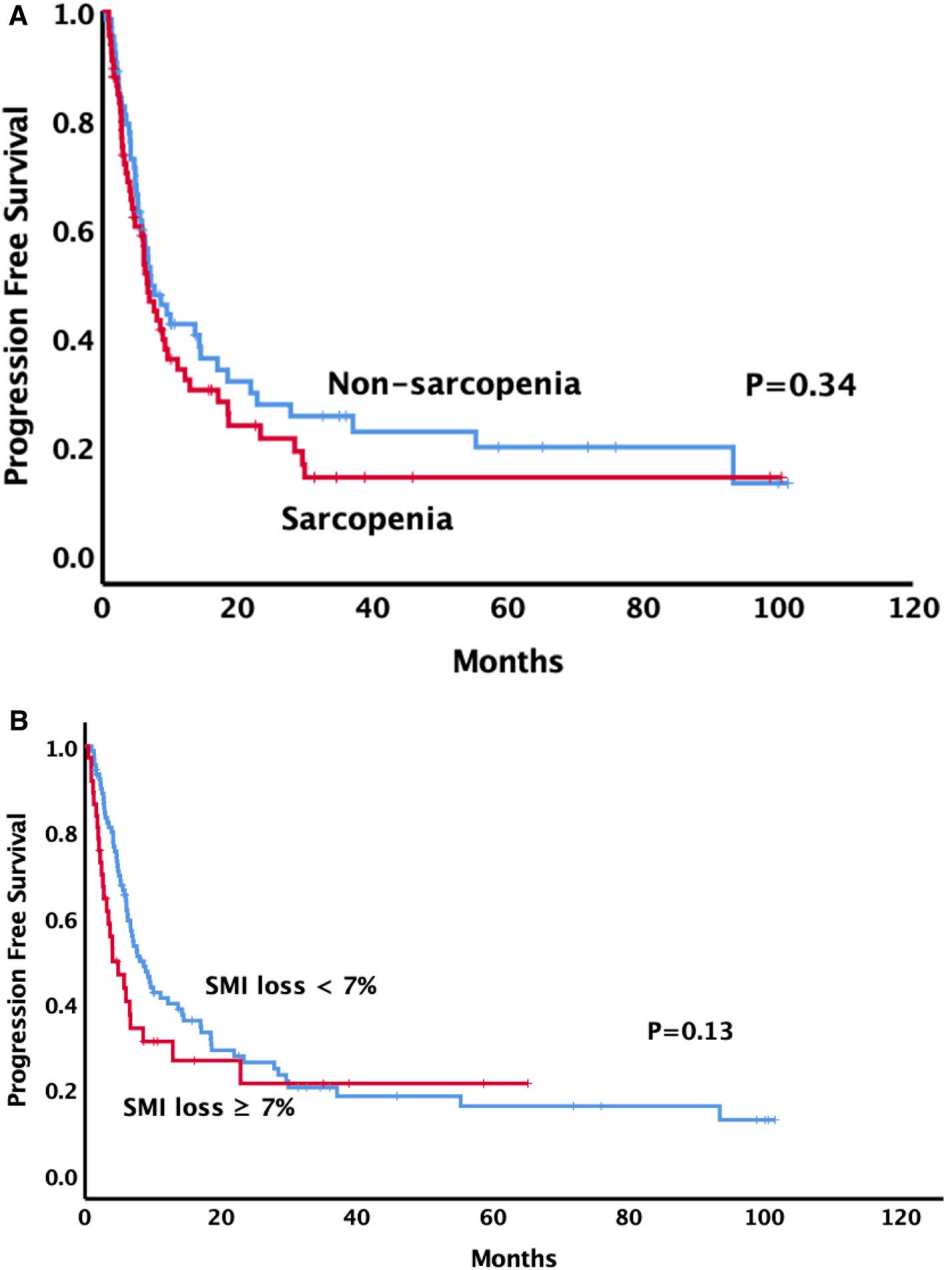
Progression free survival comparisons between (**A**) patients with and without pre-SBRT sarcopenia (**B**) patients with and without skeletal muscle loss after SBRT using Kaplan–Meier method.

With a median follow-up of 14.1 months in the entire cohort and 32.7 months in those alive, 102 deaths were observed at the time of the analysis. Thirteen patients with sarcopenia were alive, with a median survival of 16.1 months, while 22 patients without sarcopenia were alive, with a median survival of 24.0 months. The 1- and 2-year OS in patients with sarcopenia were 60.2% and 44%, respectively, compared with 64.7% and 51.8% in patients without sarcopenia (p = 0.04) (Fig. 3a). The 1- and 2-year PFS in patients with sarcopenia were 34.1% and 21.5%, respectively, compared with 42.5% and 27.7% in patients without sarcopenia (p = 0.34) (Fig. 4a). The median survival was 6.3 months and 31.4 months in patients with and without SMI loss ≥ 7%, respectively. The 1- and 2-year OS in patients with SMI loss ≥ 7% were 31.9% and 20.3%, respectively, compared with 74.9% and 59.3% in patients without SMI loss ≥ 7% (p =  < 0.001) (Fig. 3b). The 1- and 2-year PFS in patients with SMI loss ≥ 7% were 31.1% and 21.3%, respectively, compared with 41.3% and 26.2% in patients without SMI loss ≥ 7% (p = 0.13) (Fig. 4b).

### Prognostic factors

On univariate analysis, independent survival predictors were the presence of pre-SBRT sarcopenia, SMI loss, biological effective dose, ECOG, presence of extrahepatic metastasis, neutrophil-to-lymphocyte ratio (NLR), albumin-bilirubin score, tumor size, multiple tumors, and macrovascular invasion. On multivariate analysis, independent survival predictors were SMI loss, presence of extrahepatic metastasis, NLR, and multiple tumors, in which there was only a statistically significant trend for the presence of pre-SBRT sarcopenia and tumor size (Table 2).

**Table 2:** Uni- and multi-variate analysis for overall survival.

Variable	Univariate		Multivariate	
	HR (95% CI)	p	HR (95% CI)	p
Pre-SBRT sarcopenia	1.50 (1.01–2.22)	0.04	1.61 (0.99–2.61)	0.05
SMI loss ≥ 7%	3.24 (2.11–4.99)	< 0.001	1.96 (1.15–3.33)	0.01
Age > 60	1.20 (0.80–1.79)	0.39		
Sex male vs. female	0.94 (0.59–1.49)	0.76		
ECOG ≥ 2	3.38 (1.90–5.99)	< 0.001	1.59 (0.80–3.15)	0.19
AFP ≥ 200 ng/ml	1.45 (0.97–2.18)	0.07	1.44 (0.87–2.39)	0.16
ALBI (per 0.01-unit increase)	1.005 (1.002–1.009)	0.003	0.999 (0.995–1.004)	0.77
NLR ≥ 2.5	2.27 (1.47–3.49)	< 0.001	1.79 (1.07–3.00)	0.03
Prior treatment	1.00 (0.67–1.50)	1.00		
Multiple tumors	2.19 (1.46–3.30)	< 0.001	2.19 (1.30–3.71)	0.003
Tumor size ≥ 5 cm	2.12 (1.42–3.16)	< 0.001	1.57 (0.95–2.59)	0.08
Macrovascular invasion	1.92 (1.27–2.92)	0.002	1.26 (0.76–2.07)	0.37
Extrahepatic metastasis	6.65 (3.90–11.32)	< 0.001	3.47 (1.79–6.73)	< 0.001
BED (per 1 Gy increase)	0.985 (0.976–0.995)	0.003	0.998 (0.985–1.010)	0.69

SBRT: stereotactic body radiotherapy; SMI: skeletal muscle index; ECOG: Eastern Cooperative Oncology Group; AFP: alpha fetal protein; ALBI: albumin-bilirubin; NLR: neutrophil lymphocyte ratio; BED: biological effective dose; HR: hazard ratio; CI: confidence interval.

To clarify the prognostic significance of SMI loss, separate Cox models were performed according to the absence and presence of pre-SBRT sarcopenia. Multivariate analysis showed that SMI loss ≥ 7% remained an independent survival predictor in patients with sarcopenia rather than in patients without sarcopenia (Table 3). The results were similar regardless of the different definitions applied (eTables 2–7 in the Supplement).

**Table 3:** Multivariate analysis for overall survival according to the presence of pre-SBRT sarcopenia.

Variable	Sarcopenia		Nonsarcopenia	
	HR (95% CI)	p	HR (95% CI)	p
SMI loss ≥ 7%	3.06 (1.22–7.63)	0.02	1.73 (0.72–4.13)	0.22
ECOG ≥ 2	1.39 (0.48–4.04)	0.55	3.58 (1.16–11.01)	0.03
Extrahepatic metastasis	2.42 (0.73–8.02)	0.15	3.36 (1.36–8.31)	0.009
AFP ≥ 200 ng/ml	3.30 (1.51–7.20)	0.003	0.90 (0.37–2.18)	0.81
ALBI (per 0.01-unit increase)	1.000 (0.990–1.006)	0.93	0.998 (0.991–1.005)	0.52
NLR ≥ 2.5	3.61 (1.60–8.14)	0.002	1.50 (0.68–3.29)	0.31
Multiple tumors	3.66 (1.47–9.13)	0.005	2.44 (1.08–5.54)	0.03
Tumor size ≥ 5 cm	0.87 (0.41–1.86)	0.72	2.40 (1.16–4.97)	0.02
Macrovascular invasion	1.93 (0.88–4.20)	0.10	1.05 (0.46–2.36)	0.92
Extrahepatic metastasis	2.42 (0.73–8.02)	0.15	3.36 (1.36–8.31)	0.009
BED (per 1 Gy increase)	1.002 (0.984–1.020)	0.83	0.990 (0.972–1.007)	0.25

SBRT: stereotactic body radiotherapy; SMI: skeletal muscle index; ECOG: Eastern Cooperative Oncology Group; AFP: alpha fetal protein; ALBI: albumin-bilirubin; NLR: neutrophil lymphocyte ratio; BED: biological effective dose; HR: hazard ratio; CI: confidence interval.

Logistic regression demonstrated that patients who were female, older, or had pre-SBRT non-sarcopenia, higher NLR, and larger tumors were more likely to develop SMI loss ≥ 7% after SBRT (Table 4).

**Table 4:** Factors associated with the occurrence of SMI loss.

Variable	OR (95%CI)	P-value
Pre-SBRT sarcopenia	0.24 (0.06–0.94)	0.04
BMI ≥ 24	0.48 (0.15–1.53)	0.21
Age (per 1-unit increase)	1.06 (1.01–1.12)	0.03
Sex female	26.70 (3.37–211.37)	0.002
ECOG ≥ 2	1.90 (0.42–8.63)	0.40
AFP ≥ 200 ng/ml	3.40 (0.90–12.88)	0.07
ALBI (per 0.01-unit increase)	1.020 (0.986–1.054)	0.25
Albumin (per 0.1-unit increase)	1.08 (0.76–1.52)	0.68
NLR (per 0.1-unit increase)	1.03 (1.00–1.05)	0.02
Child–Pugh A	3.43 (0.45–26.49)	0.24
Prior treatment	0.98 (0.32–3.02)	0.98
Multiple tumors	3.43 (0.99–11.90)	0.05
Tumor size ≥ 5 cm	6.32 (1.61–24.88)	0.008
Extrahepatic metastasis	0.87 (0.20–3.85)	0.86
Macrovascular invasion	1.80 (0.54–6.04)	0.34
BED (per 1 Gy increase)	0.999 (0.972–1.027)	0.96

SMI: skeletal muscle index; SBRT: stereotactic body radiotherapy; BMI: body mass index; ECOG: Eastern Cooperative Oncology Group; AFP: alpha fetal protein; ALBI: albumin-bilirubin; NLR: neutrophil lymphocyte ratio; BED: biological effective dose; OR: odds ratio; CI: confidence interval.

### Toxicity

One patient without adequate follow-up was excluded from the toxicity analysis. Thirteen patients had missing values for the post-SBRT CP score. For patients with evaluable data, pre-SBRT sarcopenia was not correlated with the incidence of CP score increase by ≥ 2 within 3 months, in the absence of disease progression (15.9% and 12.8% in patients with and without pre-SBRT sarcopenia, respectively; p = 0.67). However, more patients with SMI loss ≥ 7% experienced an increase in CP score by ≥ 2 compared to their counterparts (38.1% and 7.1% in patients with and without SMI loss ≥ 7%, respectively; p = 0.001).

## Discussion

The impact of sarcopenia on HCC patients receiving surgical, locoregional or systemic managements has been explored increasingly^32^. Most literatures indicated sarcopenia before any HCC treatments portends poor OS despite miscellaneous population and assessment tools. The link between sarcopenia and other clinical outcomes was not universally reported. A metanalysis of 13 studies suggested a strong association between sarcopenia and all-cause mortality as well as tumor recurrence in HCC patients^16^. Another metanalysis of 6 studies emphasizing HCC patients treated with hepatectomy showed preoperative sarcopenia has negative correlation with OS and disease-free survival^33^. Although growing body of evidence regarding sarcopenia in HCC has been published, there are only a small number of research examining sarcopenia in patients undergoing RT. Shiba et al.^34^ found that the presence of sarcopenia was not a predictor of OS and PFS in the analysis of 68 HCC patients treated with carbon ion. To the best of our knowledge, the present study is the first to evaluate sarcopenia in patients who underwent SBRT for HCC. Our main findings are pre-SBRT sarcopenia is associated with poor OS and SMI loss ≥ 7% after SBRT constitutes an independent and stronger predictor of OS in patients with HCC, especially in patients with pre-SBRT sarcopenia. As sarcopenia is seldom used as a parameter for prognostic evaluation, our results raise the values of sarcopenia status in clinical practice.

Our current study showed that the main prognosticator for OS was muscle volume loss rather than the presence of sarcopenia before treatment. However, most studies have revealed that sarcopenia contributes to worse survival in cancer patients^7,11,12^. This may be related to the lack of analysis of the impact of muscle volume changes which leads to an emphasis on baseline sarcopenia rather than muscle change. We believe that the detrimental effect of OS might be mainly caused by sarcopenia deterioration rather than pre-SBRT sarcopenia status. Few studies have shown results similar to our findings. Lee et al.^10^ found that SMI loss during concurrent chemoradiotherapy of > 10.0% per 150 days was independently associated with poorer OS in patients with locally advanced cervical cancer, while pretreatment sarcopenia was not an independent poor prognostic factor. A Korean study^35^ in which patients with HCC undergoing RT were analyzed, did not evaluate the effect of muscle change on survival; however, newly developed sarcopenia after RT was found to significantly affect survival among patients without pre-RT sarcopenia. This also implies the importance of sarcopenia deterioration.

Furthermore, SMI loss has a much more pronounced OS impact on patients with pre-SBRT sarcopenia, which might be explained by the extent of drawing from baseline physiological reserve. For patients with adequate muscle reserve, such as those without pre-SBRT sarcopenia, the remaining muscle volume could still maintain essential function even when significant muscle loss occurs, which indicates that preservation of skeletal muscle mass after SBRT is crucial in a specific subgroup.

The pathogenesis of sarcopenia which leads to increased mortality in patients with cancer is not fully understood. Cancer-related sarcopenia is a multifactorial process that involves physical inactivity, malnutrition, and cytokine-mediated inflammation^6^. These components affect each other in a sophisticated manner. Muscle waste is driven partly by a procatabolic state invoked by proinflammatory cytokines, such as interleukin-6 and tumor necrosis factor, which are directly released by tumor cells or produced by the host’s immune response^36^. As skeletal muscle has been identified as a secretory organ^37^, progressive muscle breakdown accelerates the loss of metabolic and immune regulation. This, in turn, further enhances generalized inflammation that facilitates tumor aggressiveness and decreases secretion of certain factors (myokines) that promote protein synthesis and maintain homeostasis. Thus, the mutually reinforcing cycle puts patients at higher mortality risk.

In accordance with other reports, NLR was strongly associated with OS in our study. NLR has been used as a biomarker of systemic inflammation and was recently noted as a survival predictor in cancer patients^38,39^. In contrast to the few previous studies demonstrating the close correlation between NLR and baseline sarcopenia^40,41^, we found that patients with higher NLR tended to develop SMI loss instead of pre-SBRT sarcopenia. It reflects not only the correlation of systemic inflammation with increased metabolism but also that patients with an underlying pro-inflammatory state seem to be vulnerable to internal and external stimuli, resulting in further muscle depletion. Our multivariate analysis revealed that SMI loss had a higher hazard ratio than NLR. This might indicate that SMI loss surpasses the NLR as a survival predictor. Given that SMI loss arises from the combinatorial effect of systemic inflammation and malnutrition, it may give us a detailed picture of the dynamic interplay.

CP score increase by ≥ 2 after RT is regarded as an indicator of radiation-induced liver injury and is associated with patient survival^42^. Our study showed that SMI loss was more correlated with the CP score increase by ≥ 2 than with the presence of pre-SBRT sarcopenia. We speculate that SMI loss may simply be a consequence of the hepatic functional impairment induced by radiation. Another speculation is that the local inflammation caused by muscle volume change itself may have a systemic effect that influences the patients’ tolerance toward treatment, resulting in toxicity disparity. The causal relationship between SMI loss and toxicity was difficult to determine in our study. Nevertheless, this suggests that patients who are prone to SMI loss should receive great attention regarding the development of liver toxicity before initiating RT.

The incidence of sarcopenia varies among studies depending on the population investigated and the cutoff values used. Differences in races/ethnicities and populations may lead to misclassification of patients if inappropriate cutoff values are used. Given that there is no uniformly agreed definition of SMI, we finally chose the Korean definition to stratify our patients for similar physiques. Based on our analysis, we identified a population-specific SMI threshold of 50.5 cm^2^/m^2^ for men and 30.9 cm^2^/m^2^ for women to predict survival. These cutoff values are nearly identical to those reported by Koreans (SMI ≤ 49 cm^2^/m^2^ for men and ≤ 31 cm^2^/m^2^ for women). When other predefined specific SMIs were applied, SMI loss ≥ 7% was consistently a strong prognostic factor, while pre-sarcopenia showed inconsistent statistical significance (eTables 2–7 in the Supplement). Through robust statistics, we demonstrate that the characteristics of SMI change are its steadiness in any circumstance, regardless of the definitions applied. Unlike baseline sarcopenia status, SMI change would not be limited by races/ethnicities and populations and probably became a useful parameter in prognostic evaluation and decision making.

Acquisition of muscle volumes and changes is not a difficult task in HCC patients receiving SBRT since CT is almost routinely performed at simulation and for staging and disease monitoring. Early detection of sarcopenia in HCC patents reminds radiation oncologists to start multidimensional approach employing exercise and nutritional supplementation for reversal of muscle wasting. Moreover, radiation oncologists can precisely select suitable patients for SBRT and apply more strict dose constraints for susceptible patients to minimize liver toxicity.

Of note, research on sarcopenia in oncologic field is still in its infancy, especially in HCC patients treated with RT. Future works can focus on improvement of diagnosis, understanding of molecular and cellular mechanisms, identification of biomarkers, development of effective pharmacologic agents and artificial intelligence assisted radiomics. Additional prospective investigations are required to determine the impact of muscle loss on HCC.

## Limitations

This study has some limitations. Due to the retrospective nature of the study, we were unable to collect all confounding factors. We also did not evaluate muscle functions, such as grip strength and walking speed. The European Working Group on Sarcopenia in Older People guideline^43^ and the Asian Working Group for Sarcopenia guideline^44^, both incorporate assessments of muscle mass and function for sarcopenia diagnosis. This limitation exists in most studies on sarcopenia. Future studies involving sarcopenia should consider muscle function.

## Conclusions

In conclusion, we revealed that patients with HCC with SMI loss ≥ 7% after SBRT, rather than pre-SBRT sarcopenia had worse survival and tended to develop a CP score increase by ≥ 2 within 3 months. This underscores the importance of continually assessing the muscle mass and preventing muscle wasting after SBRT. Further prospective studies are required to validate our results and to seek proper interventions to maintain muscle mass.

## Supplementary Information

Supplementary Tables.

## Data Availability

The datasets generated during and/or analyzed in this research are available from the author upon reasonable request.
