# Supplementary material for: Significant muscle loss after stereotactic body radiotherapy predicts worse survival in patients with hepatocellular carcinoma
Source: Sci Rep. 2022 Nov 9;12:19100. doi: 10.1038/s41598-022-21443-6 (PMC9646692; doi:10.1038/s41598-022-21443-6)
Supplement: Supplementary file 1 — Supplementary Tables. [file 41598_2022_21443_MOESM1_ESM.doc]

Table S1: Clinicopathological characteristics of patients with SMI loss ≥ 7% vs. SMI loss < 7%

|  | No. (%) |  |  |  |  | |
| --- | --- | --- | --- | --- | --- | --- |
| Variable | Overall  N =137 | SMI loss ≥ 7%  N = 39 | SMI loss < 7%  N = 98 | P-value |  | |
| Age, year |  |  |  |  |  | |
| Mean (SD)* | 63.8 (12.7) | 63.8 (12.8) | 63.9 (12.5) | 0.96 |  | |
| ≤60 | 52 (38.0) | 15 (38.5) | 37 (37.8) | 0.94 |  | |
| >60 | 85 (62.0) | 24 (61.5) | 61 (62.2) |  |  | |
| Sex |  |  |  | 0.08 |  | |
| Male | 106 (77.8) | 34 (87.2) | 72 (73.5) |  |  | |
| Female | 31 (22.2) | 5 (12.8) | 26 (26.5) |  |  | |
| Liver disease |  |  |  | 0.22 |  | |
| HBV | 71 (51.8) | 24 (61.5) | 47 (48.0) |  |  | |
| HCV | 36 (26.3) | 9 (23.1) | 27 (27.6) |  |  | |
| HBV and HCV | 8 (5.8) | 0 (0) | 8 (8.2) |  |  | |
| Non-virus | 22 (16.1) | 6 (15.4) | 16 (16.3) |  |  | |
| ECOG |  |  |  | 0.03 |  | |
| 0-1 | 119 (86.9) | 30 (76.9) | 89 (90.8) |  |  | |
| 2 | 18 (13.1) | 9 (23.1) | 9 (9.2) |  |  | |
| AFP, ng/ml |  |  |  | 0.04 |  | |
| ≤200 | 79 (57.7) | 18 (46.2) | 61 (62.6) |  |  | |
| >200 | 51 (37.2) | 20 (51.3) | 31 (31.6) |  |  | |
| missing | 7 (5.1) | 1 (2.6) | 6 (6.1) |  |  | |
| ALBI score |  |  |  | 0.005 |  | |
| Mean (SD)* | -2.42 (0.57) | -2.20 (0.61) | -2.50 (0.53) |  |  | |
| Child-Pugh Class |  |  |  | 0.12 |  | |
| A | 110 (80.3) | 27 (69.2) | 83 (84.7) |  |  | |
| B | 23 (16.8) | 10 (25.6) | 13 (13.3) |  |  | |
| C | 4 (2.9) | 2 (5.1) | 2 (2.0) |  |  | |
| Albumin |  |  |  | 0.04 |  | |
| Mean (SD)* | 3.70 (0.56) | 3.55 (0.58) | 3.77 (0.54) |  |  | |
| NLR |  |  |  | 0.02 |  | |
| ≤2.5 | 57 (41.6) | 11 (28.2) | 46 (46.9) |  |  | |
| >2.5 | 62 (45.3) | 24 (61.5) | 38 (38.8) |  |  | |
| Missing | 18 (13.1) | 4 (10.3) | 14 (14.3) |  |  | |
| Prior treatment |  |  |  | 0.75 |  | |
| Yes | 85 (62) | 25 (64.1) | 60 (61.2) |  |  | |
| No | 52 (38) | 14 (35.9) | 38 (38.8) |  |  | |
| No. of tumor |  |  |  | 0.03 |  | |
| Multiple | 78 (56.9) | 28 (71.8) | 50 (51.0) |  |  | |
| Single | 59 (43.1) | 11 (28.2) | 48 (49.0) |  |  | |
| Tumor size, cm |  |  |  | 0.001 |  | |
| ≤5 | 67 (48.9) | 10 (25.6) | 57 (58.2) |  |  |  |
| >5 | 70 (51.1) | 29 (74.4) | 41 (41.8) |  |  | |
| Macrovascular invasion |  |  |  | 0.03 |  | |
| Yes | 47 (34.3) | 19 (48.7) | 28 (28.6) |  |  | |
| No | 90 (65.7) | 20 (51.3) | 70 (71.4) |  |  | |
| Extrahepatic metastasis |  |  |  | 0.01 |  | |
| Yes | 27 (19.7) | 13 (33.3) | 14 (14.3) |  |  | |
| No | 110 (80.3) | 26 (66.7) | 84 (85.7) |  |  | |
| BMI |  |  |  | 0.05 |  | |
| Mean (SD)* | 24.7 (4.1) | 23.6 (4.1) | 25.1 (4.1) |  |  | |
| SMI |  |  |  | 0.84 |  | |
| Mean (SD)* | 44.9 (8.8) | 45.1 (8.2) | 44.8 (9.0) |  |  | |
| BED, Gy |  |  |  | 0.004 |  | |
| Mean (SD)* | 87.4 (21.9) | 79.0 (18.5) | 90.8 (22.3) |  |  | |

*Abbreviations:* SMI= skeletal muscle index; HBV = hepatitis B virus; HCV= hepatitis C virus; ECOG = Eastern Cooperative Oncology Group; AFP = alpha fetal protein; ALBI= albumin-bilirubin; NLR= neutrophil lymphocyte ratio; BMI= body mass index; BED = biological effective dose; SD = standard deviation. *t-test

Table S2: Uni- and multi-variate analysis for OS based on sarcopenia definition proposed by Martin et al.

|  | Univariate | | Multivariate | |
| --- | --- | --- | --- | --- |
| Variable | HR (95% CI) | p | HR (95% CI) | p |
| Pre-SBRT sarcopenia | 1.41 (0.95-2.11) | 0.09 | 1.40 (0.89-2.20) | 0.15 |
| SMI loss≥7% | 3.24 (2.11-4.99) | <0.001 | 2.04 (1.19-3.51) | 0.009 |
| Age>60 | 1.20 (0.80-1.79) | 0.39 |  |  |
| Sex male vs. female | 0.94 (0.59-1.49) | 0.76 |  |  |
| ECOG≥2 | 3.38 (1.90-5.99) | <0.001 | 1.64 (0.84-3.22) | 0.15 |
| AFP ≥200 ng/ml | 1.45 (0.97-2.18) | 0.07 | 1.36 (0.82-2.27) | 0.24 |
| ALBI (per 0.01-unit increase) | 1.005 (1.002-1.009) | 0.003 | 0.999 (0.995-1.003) | 0.67 |
| NLR≥2.5 | 2.27 (1.47-3.49) | <0.001 | 1.76 (1.06-2.92) | 0.03 |
| Prior Treatment | 1.00 (0.67-1.50) | 1.00 |  |  |
| Multiple tumors | 2.19 (1.46-3.30) | <0.001 | 2.08 (1.24-3.50) | 0.006 |
| Tumor size≥5 cm | 2.12 (1.42-3.16) | <0.001 | 1.68 (1.03-2.74) | 0.04 |
| Macrovascular invasion | 1.92 (1.27-2.92) | 0.002 | 1.19 (0.72-1.96) | 0.49 |
| Extrahepatic metastasis | 6.65 (3.90-11.32) | <0.001 | 3.48 (1.80-6.74) | <0.001 |
| BED (per 1Gy increase) | 0.985 (0.976-0.995) | 0.003 | 0.995 (0.983-1.007) | 0.37 |

*Abbreviations:* OS = overall survival; SBRT = stereotactic body radiotherapy; SMI = skeletal muscle index; ECOG = Eastern Cooperative Oncology Group; AFP = alpha fetal protein; ALBI = albumin-bilirubin; NLR = neutrophil lymphocyte ratio; BED = biological effective dose; HR = hazard ratio; CI = confidence interval.

Table S3: Multivariate analysis for OS according to the presence of pre-SBRT sarcopenia defined by Martin et al.

|  | Sarcopenia | | Nonsarcopenia | |
| --- | --- | --- | --- | --- |
| Variable | HR (95% CI) | p | HR (95% CI) | p |
| SMI loss≥7% | 2.58 (1.03-6.47) | 0.04 | 1.58 (0.67-3.71) | 0.30 |
| ECOG≥2 | 1.53 (0.66-3.50) | 0.32 | 1.45 (0.37-5.69) | 0.59 |
| AFP ≥200 ng/ml | 1.71 (0.83-3.55) | 0.15 | 1.16 (0.49-2.76) | 0.74 |
| ALBI (per 0.01-unit increase) | 1.004 (0.996-1.012) | 0.31 | 0.999 (0.993-1.005) | 0.65 |
| NLR≥2.5 | 1.84 (0.93-3.61) | 0.08 | 1.70 (0.74-3.90) | 0.21 |
| Multiple tumors | 2.80 (1.31-5.98) | 0.008 | 2.20 (0.83-5.86) | 0.12 |
| Tumor size≥5 cm | 0.81 (0.39-1.68) | 0.57 | 2.72 (1.18-6.26) | 0.02 |
| Macrovascular invasion | 1.54 (0.73-3.24) | 0.26 | 1.27 (0.58-2.81) | 0.55 |
| Extrahepatic metastasis | 1.98 (0.71-5.52) | 0.19 | 3.50 (1.30-9.42) | 0.01 |
| BED (per 1Gy increase) | 0.993 (0.979-1.008) | 0.34 | 0.993 (0.969-1.019) | 0.61 |

*Abbreviations:* OS = overall survival; SBRT = stereotactic body radiotherapy; SMI = skeletal muscle index; ECOG = Eastern Cooperative Oncology Group; AFP = alpha fetal protein; ALBI = albumin-bilirubin; NLR = neutrophil lymphocyte ratio; BED = biological effective dose; HR = hazard ratio; CI = confidence interval.

Table S4: Uni- and multi-variate analysis for OS based on sarcopenia definition proposed by Prado et al.

|  | Univariable | | Multivariable | |
| --- | --- | --- | --- | --- |
| Variable | HR (95% CI) | p | HR (95% CI) | p |
| Pre-SBRT sarcopenia | 1.32 (0.85-2.04) | 0.22 | 1.15 (0.69-1.92) | 0.60 |
| SMI loss≥7% | 3.24 (2.11-4.99) | <0.001 | 1.94 (1.14-3.29) | 0.01 |
| Age>60 | 1.20 (0.80-1.794) | 0.39 |  |  |
| Sex male vs. female | 0.94 (0.59-1.49) | 0.76 |  |  |
| ECOG≥2 | 3.38 (1.90-6.00) | <0.001 | 1.76 (0.88-3.53) | 0.11 |
| AFP≥200 ng/ml | 1.45 (0.97-2.18) | 0.07 | 1.35 (0.81-2.25) | 0.25 |
| ALBI (per 0.01-unit increase) | 1.005 (1.002-1.009) | 0.003 | 0.999 (0.995-1.003) | 0.68 |
| NLR≥2.5 | 2.27 (1.47-3.49) | <0.001 | 1.72 (1.03-2.86) | 0.04 |
| Prior Treatment | 1.00 (0.67-1.50) | 1.00 |  |  |
| Multiple tumors | 2.19 (1.46-3.30) | <0.001 | 2.13 (1.26-3.58\) | 0.004 |
| Tumor size≥5 cm | 2.12 (1.42-3.16) | <0.001 | 1.72 (1.04-2.83) | 0.03 |
| Macrovascular invasion | 1.92 (1.27-2.92) | 0.002 | 1.22 (0.74-2.03) | 0.44 |
| Extrahepatic metastasis | 6.65 (3.90-11.32) | <0.001 | 3.26 (1.68-6.35) | <0.001 |
| BED | 0.985 (0.976-0.995) | 0.003 | 0.995 (0.983-1.007) | 0.43 |

*Abbreviations:* OS = overall survival; SBRT = stereotactic body radiotherapy; SMI = skeletal muscle index; ECOG = Eastern Cooperative Oncology Group; AFP = alpha fetal protein; ALBI = albumin-bilirubin; NLR = neutrophil lymphocyte ratio; BED = biological effective dose; HR = hazard ratio; CI = confidence interval.

Table S5: Multivariate analysis for OS according to the presence of pre-SBRT sarcopenia defined by Prado et al.

|  | Sarcopenia | | Nonsarcopenia | |
| --- | --- | --- | --- | --- |
| Variable | HR (95% CI) | p | HR (95% CI) | p |
| SMI loss≥7% | 2.15 (1.09-4.25) | 0.03 | 1.65 (0.58-4.69) | 0.35 |
| ECOG≥2 | 1.29 (0.57-2.94) | 0.54 | 3.46 (0.61-19.58) | 0.16 |
| AFP≥200 ng/ml | 1.42 (0.78-2.57) | 0.25 | 1.16 (0.39-3.52) | 0.79 |
| ALBI | 0.998 (0.992-1.003) | 0.38 | 1.002 (0.993-1.011) | 0.62 |
| NLR≥2.5 | 1.52(0.83-2.81) | 0.18 | 2.76 (0.92-8.33) | 0.07 |
| Multiple tumors | 1.77 (0.96-3.26) | 0.07 | 3.69 (0.85-16.00) | 0.08 |
| Tumor size≥5 cm | 1.21 (0.65-2.25) | 0.55 | 3.30 (1.23-8.90) | 0.02 |
| Macrovascular invasion | 1.71 (0.91-3.26) | 0.10 | 0.79 (0.28-2.29) | 0.67 |
| Extrahepatic metastasis | 5.31 (2.06-13.74) | 0.001 | 2.38 (0.89-6.60) | 0.10 |
| BED (per 1Gy increase) | 0.987 (0.973-1.002) | 0.09 | 1.008 (0.979-1.038) | 0.59 |

*Abbreviations:* OS = overall survival; SBRT = stereotactic body radiotherapy; SMI = skeletal muscle index; ECOG = Eastern Cooperative Oncology Group; AFP = alpha fetal protein; ALBI = albumin-bilirubin; NLR = neutrophil lymphocyte ratio; BED = biological effective dose; HR = hazard ratio; CI = confidence interval.

Table S6: Uni- and multi-variate analysis for OS based on sarcopenia definition proposed by Sakurai et al.

|  | Univariable | | Multivariable | |
| --- | --- | --- | --- | --- |
| Variable | HR (95% CI) | p | HR (95% CI) | p |
| Pre-SBRT sarcopenia | 1.38 (0.92-2.09) | 0.12 | 1.51 (0.94-2.42) | 0.09 |
| SMI loss≥7% | 3.24 (2.11-4.99) | <0.001 | 2.02 (1.19-3.42) | 0.01 |
| Age>60 | 1.20 (0.80-1.80) | 0.39 |  |  |
| Sex male vs. female | 0.94 (0.59-1.49) | 0.76 |  |  |
| ECOG≥2 | 3.38 (1.90-5.99) | <0.001 | 1.69 (0.87-3.29) | 0.12 |
| AFP≥200 ng/ml | 1.45 (0.97-2.18) | 0.07 | 1.33 (0.80-2.21) | 0.27 |
| ALBI (per 0.01-unit increase) | 1.005 (1.002-1.009) | 0.003 | 0.999 (0.995-1.003) | 0.59 |
| NLR≥2.5 | 2.27 (1.47-3.49) | <0.001 | 1.86 (1.11-3.13) | 0.02 |
| Prior Treatment | 1.00 (0.67-1.50) | 1.00 |  |  |
| Multiple tumors | 2.19 (1.46-3.30) | <0.001 | 2.21 (1.31-3.75) | 0.003 |
| Tumor size≥5 cm | 2.12 (1.42-3.16) | <0.001 | 1.65 (1.00-2.70) | 0.05 |
| Macrovascular invasion | 1.92 (1.27-2.92) | 0.002 | 1.20 (0.73-1.97) | 0.48 |
| Extrahepatic metastasis | 6.65 (3.90-11.32) | <0.001 | 3.44 (1.77-6.66) | <0.001 |
| BED (per 1Gy increase) | 0.985 (0.976-0.995) | 0.003 | 0.996 (0.984-1.008) | 0.53 |

*Abbreviations:* OS = overall survival; SBRT = stereotactic body radiotherapy; SMI = skeletal muscle index; ECOG = Eastern Cooperative Oncology Group; AFP = alpha fetal protein; ALBI = albumin-bilirubin; NLR = neutrophil lymphocyte ratio; BED = biological effective dose; HR = hazard ratio; CI = confidence interval.

Table S7: Multivariate analysis for OS according to the presence of pre-SBRT sarcopenia defined by Sarukai et al.

|  | Sarcopenia | | Nonsarcopenia | |
| --- | --- | --- | --- | --- |
| Variable | HR (95% CI) | p | HR (95% CI) | p |
| SMI loss≥7% | 4.85 (1.42-16.53) | 0.01 | 1.73 (0.87-3.43) | 0.12 |
| ECOG≥2 | 1.51 (0.36-6.39) | 0.57 | 2.05 (0.81-5.18) | 0.13 |
| AFP≥200 ng/ml | 2.48 (0.86-7.18) | 0.09 | 1.22 (0.62-2.41) | 0.57 |
| ALBI (per 0.01-unit increase) | 0.998 (0.990-1.006) | 0.62 | 0.999 (0.993-1.004) | 0.61 |
| NLR≥2.5 | 4.37 (1.60-11.93) | 0.004 | 1.43 (0.74-2.75) | 0.29 |
| Multiple tumors | 4.23 (1.15-15.65) | 0.03 | 2.309 (1.15-4.64) | 0.02 |
| Tumor size≥5 cm | 0.67 (0.24-1.88) | 0.44 | 1.93 (1.06-3.53) | 0.03 |
| Macrovascular invasion | 2.10 (0.71-6.17) | 0.18 | 1.29 (0.67-2.47) | 0.45 |
| Extrahepatic metastasis | 2.30 (0.48-10.97) | 0.30 | 3.75 (1.70-8.26) | 0.001 |
| BED (per 1Gy increase) | 0.998 (0.978-1.018) | 0.82 | 0.995 (0.980-1.010) | 0.53 |

*Abbreviations:* OS = overall survival; SBRT = stereotactic body radiotherapy; SMI = skeletal muscle index; ECOG = Eastern Cooperative Oncology Group; AFP = alpha fetal protein; ALBI = albumin-bilirubin; NLR = neutrophil lymphocyte ratio; BED = biological effective dose; HR = hazard ratio; CI = confidence interval.
